# Evaluating autobiographical skills and their relationship with suggestibility in children: development and validation of the Children Recalling Autobiographical Memory

**DOI:** 10.3389/fpsyg.2024.1321305

**Published:** 2024-01-23

**Authors:** Monia Vagni, Valeria Giostra, Luca Simione

**Affiliations:** ^1^Department of Philosophy, Social Sciences, Humanities and Education, University of Perugia, Perugia, Italy; ^2^Department of Humanities, University of Urbino, Urbino, Italy; ^3^Dipartimento di Scienze Umane e Sociali Internazionali, UNINT, Università degli Studi Internazionali, Rome, Italy; ^4^Istituto fdi Scienze e Tecnologie della Cognizione, CNR, Rome, Italy

**Keywords:** narrative skills, suggestibility, misleading questions, autobiographical memory, children

## Abstract

**Introduction:**

Autobiographical narrative skills and resistance to suggestibility factors are central aspects in children’s testimony. While the assessment of suggestibility relies on standardized questionnaire, no such an instrument exists to reliably assess autobiographical skills in children. This aspect is further important when considering that the development of such skills seems to be related to the suggestibility, that is, suggestibility would be reduced in presence of higher autobiographical skills. However, no direct test of this relationship is available in literature, also due to the lack of quantitative instruments for assessing autobiographical skills.

**Methods:**

To fulfill both these methodological and theoretical issues, in this study a new tool was validated to measure the main autobiographical narrative skills (Where, What, When, Who, and How) in relation to both Retrospective Memory and Prospective Memory: the Children Recalling Autobiographical Memory (CRAM). We recruited a sample of 321 children aged 7–16 years.

**Results and discussion:**

The result of the EFA analysis showed one-factor model, and revealed also good fit indexes and internal reliability. After validating this new tool, we further used it to test our main hypothesis, that is, children with higher autobiographical memory skills were less vulnerable to interrogative suggestibility as assessed by Gudjonsson Suggestibility Scale 2 (GSS2). A hierarchical linear regression model showed a reduction in suggestibility with age and level of autobiographical skills. Moreover, the level of such skills moderate the effect of age, such as only in presence of high or moderate level of autobiographical skills the age significantly reduces the level of suggestibility.

## 1 Introduction

Main aspects of the children’s testimony are the ability to remember autobiographical events and the resistance to suggestibility factors that can alter the original memory. Numerous studies have shown that children are more vulnerable to suggestive questions and post-event information. Children also show a tendency to provide autobiographical memories that are poorer in detail and have a faster memory decay curve (Goodman et al., 2002; Loftus, 2005; Bauer and Larkina, 2016; Gudjonsson et al., 2016; Vagni et al., 2023).

The emotionally negative events in children can decay earlier or be only partially recalled despite some cues (Jones and Pipe, 2002). The anxious and psychological activation associated with such events, especially if traumatic, would lead to greater suggestive vulnerability and confabulatory recall (Otgaar et al., 2010; Otgaar and Howe, 2018; Gudjonsson et al., 2020, 2021; Vagni et al., 2021, 2022). The re-enactment of autobiographical events requires adequate narrative skills (Nelson and Fivush, 2004).

Especially in cases of suspected sexual violence, the children’s testimony is often the only one at trial. Child witnesses often undergo evaluation by an expert who must ascertain their ability to remember autobiographical events and their ability to resist suggestibility factors. Poor autobiographical memory and suggestive vulnerability can limit or exclude children’s testimonial reliability (Lamb et al., 2018). In forensic contexts it is necessary that expert assessments derive from scientific evidence and objective measurement tools.

In this study we will first present a definition of children’s autobiographical memory and suggestibility to understand the association between these two factors. Subsequently, since there is a lack of a psychometric instrument that measures autobiographical memory and autobiographical narrative skills in children, the validation of a new instrument will be presented. Finally, it will be illustrated how autobiographical skills can intervene on levels of suggestive vulnerability.

### 1.1 Autobiographical memory and narrative skills

Autobiographical memory is a constructive process that concerns both retrospective memory and prospective memory. Previous autobiographical events allow one to imagine and predict future experiences (Schacter and Addis, 2007). During the narration of a memory the spatial and visual information of what they experienced is reactivated, processed and converted into a verbal narrative. In the process of retrieving this information various cognitive functions are involved which allow the activated visual images to be faithful to the original memory, and expressed in a clear, accurate and complete way (Rubin et al., 2003).

The model developed by Conway et al. (2004) and Conway (2009) distinguishes between working self, autobiographical memory, and episodic memory. The working self consists of the conceptual self (Conway et al., 2004) and the goal system. It is the working self that determines which knowledge derived from experience is encoded in autobiographical memory. The authors highlighted that encoding lasts for a limited time and includes sensorial-perceptual information, affects, thoughts, imaginations (Williams et al., 2008). Autobiographical memory contains autobiographical knowledge, the history of our times and episodic memories that derive from lived experience (Conway and Pleydell-Pearce, 2000; Conway, 2005). In the model of Conway (2009), autobiographical memory consists of retrospective autobiographical memory, prospective autobiographical memory, and semantic autobiographical memory.

Autobiographical episodic memory is a constructive process that allows people to flexibly extract and recombine elements of the past. A similar reconstructive process concerns prospective or future memory where previous knowledge and experiences allow us to simulate future ones (Schacter and Addis, 2007). Neuroimaging studies have highlighted how the coding and learning phases of new events affect brain areas different from those involved in the re-enactment phases. Several studies have also highlighted how it is not possible to distinguish the brain areas involved in the recall of a real memory from the production of a fantastic memory (Schacter and Addis, 2007; Paz-Alonso et al., 2008; Van de Ven et al., 2018). As suggested by several studies, the activity of recovering a real memory or imagining a future memory involves the same brain areas. This could explain how at a cognitive level there are no differences between retrospective memory and prospective memory. Indeed, the recall of past events and the imagination of future events involve the same cognitive abilities (Fan et al., 2022). In fact, prospective memory is based on personal events that the people have already experienced, and this makes planning and forecasting what will happen easier and more consistent with one’s sense of identity. According to Nelson (1986), autobiographical memory also has the function of facilitating the prediction of future events, allowing to better anticipate what will happen in the face of recurring events and facilitating defense responses.

According to Wood and Conway (2006), the emotional component also plays an important role in the encoding and reactivation of autobiographical memories in terms of intensity. The positive or negative valence can allow the preservation of vividness, a greater retention over time of the memory and the quality of the narrative structure of the memory.

The ability to tell an autobiographical event is given by the narrative skills that increase with age and allow a description of when, how, where and what happened (Nelson and Fivush, 2004). Indeed, the main narrative skills allow you to give space–time information (Nelson and Fivush, 2004), the description of what happened, who was involved and how the episode occurred (Rubin et al., 2003; Wood and Conway, 2006). According to several studies, narrative skills increase with age and with the development of intellectual and expressive abilities (Nelson and Fivush, 2004; Orbach and Lamb, 2007; Kulkofsky and Klemfuss, 2008). With age, the capacity for retrospective mnemic activation increases in children, even after a long time, which allows them to recall and associate information and provide a story that is even richer in detail (Lamb et al., 2018).

Several studies shown that even children under six had the ability to reliably remember some autobiographical events, even after 1 year (Hudson and Fivush, 1991), but it is necessary to provide a cue to retrieve information. According to Nieto et al. (2017), after the age of 4,5, preschool children show an ability to recall personal events through the cueing. In any case, the accounts of younger children tend to be more fragmented than older children (Newcombe et al., 2007). The youngest children can tell what happened, but the ability to place the event more accurately in time develops after age 10 and becomes complete and adult-like after age 14 (Hudson and Mayhew, 2009; Roberts et al., 2015; Jack et al., 2016).

Narrating repeated events can promote confidence in terms of narrative skills, to the detriment of details relating to individual episodes if they present similar characteristics. The narration of specific events can be associated with more vivid and detailed descriptions, while common events tend to be narrated for the most central elements due to a certain rigidity of the mnemic scheme (Wang et al., 2011; Roberts et al., 2015).

The knowledge and semantic understanding of what is happened facilitates its narration. For this reason, studies on children’s autobiographical memory used frequent cue words belonging to children’s language. From the age of 8, autobiographical narration appears to increase in relation to the increase in children’s semantic, linguistic and cognitive skills (Brubacher et al., 2012).

According to Kulkofsky and Klemfuss (2008), both social and cognitive processes are involved in developing adequate narrative skills. By sharing and narrating their experiences to others, children learn to attribute meaning to events and to organize the main markers of the narrative which are linguistic-semantic and temporal (not only of when it happened, but also in terms of sequentiality before and after, so as to say a linear succession of what happened; Nelson and Fivush, 2004; Kulkofsky and Klemfuss, 2008).

Security and trust in one’s memory can represent protective factors compared to factors of distortion and suggestion. Distrust Memory Syndrome is in fact one of the main factors of vulnerability to misleading information and social pressures during a forensic interrogation (Gudjonsson, 2018).

Several studies have highlighted gender differences in the production of episodic events and in the description of details. Women tend to show a greater ability to retrieve specific autobiographical events than men (Herlitz and Rehnman, 2008; Wang et al., 2011). Gender differences in episodic memory would tend to emerge early. Research has also found that, compared to men, women show greater accessibility in recalling personal experiences from early childhood (Rubin, 2000), and are more capable of recalling memories of specific events, events unique or happened only once.

### 1.2 Suggestibility and autobiographical narrative skills

Lamb et al. (2018) found that judicial interviews with children occur using lots of suggestive questions that risk to altering the reconstruction of what happened and the memory of the event. The model of suggestibility that has found wide validity in the forensic field is that Interrogative or Immediate Suggestibility of Gudjonsson and Clark (1986). When witnesses are exposed to unanswerable or misleading questions, they may show weakness due to their uncertainty, vulnerability toward authority figures and high expectations of success. Their vulnerability may increase if exposed to negative criticism, leading them to modify their responses and be more suggested (Gudjonsson, 2018; Vagni et al., 2018). Younger children generally present greater vulnerability and a lower ability to provide resistant responses and use adequate coping strategies (Maiorano and Vagni, 2020; Gudjonsson et al., 2021, 2022; Vagni et al., 2021, 2023). Males and females show the same vulnerability to interrogative suggestibility. In fact, several studies have not found gender differences in answering leading questions (Gudjonsson, 2003; Klemfuss and Olaguez, 2020).

Loftus’ studies have found that, despite the presence of a very accurate autobiographical memory, both children and adults can be induced to modify their memory trace in a suggestive way (Berkowitz and Loftus, 2018). Other studies, on the contrary, showed that having high narrative skills had a protective function with regard to external sources of suggestion. Kulkofsky and Klemfuss (2008) have showed that having high narrative skills is associated with a greater source monitoring capacity which allows children to evaluate the information being acquired and compare it with their own knowledge to carry out assessments of discrimination, coherence, plausibility in a more plastic and flexible way. High autobiographical narrative skills thus seem to be related to reduced levels of yielding to suggestive questions.

The ability to handle memory and cognitive patterns more flexibly increases with age and this seems to ensure that children are more competent in processing and managing suggestibility factors (Gudjonsson et al., 2016). In line with this, Gudjonsson et al. (2021, 2022) proposed that trust in one’s memory and the high source monitoring skill are protective and resistant factors to suggestive vulnerability.

### 1.3 Assessment of autobiographical memory skills and suggestibility in children

The ability to recall autobiographical information varies significantly in both children and adults. This variability appears to be primarily linked to individual characteristics that influence the vividness and accuracy of reactivating visuo-spatial information. In children, the development of autobiographical abilities appears to increase with age. Emotional factors also contribute significantly to this process, as lower emotional arousal tends to be associated with a weaker recall compared to situations with higher emotional arousal. Most studies on autobiographical memory have employed the cueing technique (Crovitz and Schiffman, 1974; Rubin and Schulkind, 1997; Conway et al., 1999; Congleton and Berntsen, 2018). However, it is essential to note that the cueing technique used in these studies comes with recognized limitations in the scientific community. Firstly, it is important to emphasize that the stimulus words used often lead to an excessive degree of freedom in memory retrieval, rendering the recollections less comparable to each other (Conway, 2005; Griffith et al., 2012). Some studies have attempted to address this limitation through the assignment of scores typically ranging from 0 to 3. According to Griffith et al. (2012), the criteria for inclusion or exclusion of responses are different in different studies. Nevertheless, it should be noted that these numerical values are associated with qualitative criteria rather than a purely metric approach. Consequently, this opens the possibility for evaluative discretion on the part of the examiner when assigning a specific score.

Clinical and forensic practice lacks a standardized measuring tool for autobiographical narrative skills. This was the main objective of our study aimed at validating a metric tool for measuring the individual narrative abilities of the autobiographical memory. In fact, many studies have dealt with children’s memory by evaluating the quality of the story or considering the presence/absence of some information (Goodman et al., 2002, 2010).

Most studies on autobiographical memory have used the Autobiographical Memory Test (AMT; Griffith et al., 2012), based on the original methodology developed by Williams and Broadbent (1986). In the AMT, participants are presented with a series of guide words, and are asked to produce a relevant personal memory. The memories provided are then coded based on level of specificity (Griffith et al., 2012). Most of these studies divided the cue words on the basis of positive vs. negative valence, and only a few studies also included neutral valence words (Griffith et al., 2012; Ros et al., 2018). Generally between 10 and 20 keywords were used (Liu et al., 2014). Generally studies that used AMT obtained a single factor solution. Age is an important factor in determining the ability to recall specific events from autobiographical memory. The AMT test was also applied to pre-school children demonstrating how children above the age of 4 can present an adequate autobiographical function (Nieto et al., 2017). The cue-words used by Nieto et al. (2017) concerned basic emotions that children may already know, such as joy, anger, and sadness.

There are also other techniques to evaluate autobiographical memory in children, such as those based on standardized open memory questions about the recent and more distant past concerning common autobiographical events in young children’s lives (Wang, 2004, 2008).

In the AMT procedure, participants are given few restrictions on the retrieval process and are told that the recalled event could have happened either recently or a long time ago as well as being told that the narrated event could be important or trivial (Griffith et al., 2012). The answers are coded on the basis of the following categories: (1) specific memories, (2) extended memories or those that refer to one or more days (for example “my wedding”), (3) categorical memories (school memories, or those linked to my family), (4) semantic associates, or (5) omissions or non-response.

However, no study has used a standardized metric tool to compare between children of different ages. Furthermore, studies on children’s autobiographical memory are based on their ability to recall memories associated with cue-words, but not on the measurement of the main narrative skills (What, When, Who, Where, and How) in terms of numerical assignment of their presence.

At same time, several studies have analyzed the suggestive vulnerability of children using metric and standardized tools (Gudjonsson et al., 2016, 2020, 2021, 2022), but not in association with objective measures of autobiographical memory. In forensic practice it is important to understand the relationship between autobiographical capacity and how children manage the suggestibility factors inherent in a forensic interview. For example, an earlier study by Kulkofsky and Klemfuss (2008) investigated the effect of narrative skills in reducing suggestibility, but without using standardized tools and metric measurements.

The Interrogative Suggestibility model is closely linked with the Gudjonsson Suggestibility Scale (Gudjonsson, 1997), a tool designed to gauge an individual’s susceptibility to suggestive influences in the context of learned and non-autobiographical memories (Vagni et al., 2015, 2017, 2022; Gudjonsson et al., 2020). To date, there are no studies that have verified the impact of autobiographical skills on interrogative suggestibility using metric and standardized measures Therefore, we aim to investigate whether, and to what extent, children of varying ages with strong autobiographical memory skills exhibit enhanced resistance to suggestibility factors. However, prior to embarking on this investigation, it is essential to address the existing gap in the literature pertaining to the absence of a valid and reliable metric tool for assessing autobiographical and narrative skills in children.

### 1.4 The present study

In this paper, we propose a new instrument called the Children Recalling Autobiographical Memory test (CRAM). This tool has its theoretical framework in the Conway’s model of autobiographical memory (Conway et al., 2004; Conway, 2009). The selection of cue-words derives from some words with positive and negative valence used in previous studies with children (Griffith et al., 2012; Nieto et al., 2017; Ros et al., 2018) and from the work of Pierucci et al. (2023). It is a tool composed of 20 cue words, which measures the main narrative skills with respect to retrospective and prospective autobiographical memory using the cueing technique. The selection of cue words occurred following the criteria used by Conway (2001), according to which autobiographical memories are organized in both a more general and specific sense, both for retrospective and prospective memory. Conway (2001) also used the following criteria: words that refer to commonly used objects; clichés and emotions. In choosing the cue words we also tried to identify terms that are also frequently used by children, and pertinent to children’s lives (such as: “birthday” and “school”). Indeed, autobiographical episodic memory is therefore linked to “temporally” significant events, but also to events repeated several times over time and linked to objects/people of everyday life (Conway, 2001). Several authors (Jones and Pipe, 2002; Wood and Conway, 2006; Conway, 2009) also highlighted how in addition to emotional intensity, personal memory is influenced by the emotional value experienced. The CRAM assesses children autobiographical and narrative skills in five main domains: Where, What, When, Who and How, giving rise to a simple and interpretable score from 0 to 5 for each item, according to the numbers of domains reported. In this study, we conducted the validation and reliability analysis on this new tool, and then we used it to study the relationship between autobiographical memory capacity and suggestibility in a sample of children of different ages.

Based on the reviewed literature, we formulated the following set of hypotheses:

*Hypothesis 1*: The assessment of autobiographical skills using the CRAM will demonstrate validity and reliability across children of different ages.

*Hypothesis 2*: Autobiographical memory will exhibit a negative correlation with suggestibility.

*Hypothesis 3*: Females show higher autobiographical memory scores but similar suggestive vulnerability to males. Age will demonstrate a negative correlation with suggestibility and positive relation with the CRAM score.

*Hypothesis 4*: The impact of age on suggestibility will be moderated by autobiographical memory skills, suggesting that in the presence of low autobiographical skills, age will not be significantly associated with reduced suggestibility.

## 2 Materials and methods

### 2.1 Participants and procedures

The sample included 321 participants (47.4% female; 52.6% male) recruited in several Italian schools. Participant ages ranged from 7 to 16 years old (*M* = 11.03 and *SD* = 2.49). Ninety-nine children (30.8%) were aged 7–9 years; 125 children (38.8%) were aged 10–12 years, and 97 children (30.2%) were 13–16 years. Data collection took place between February and May 2023 and the participants were met after their parents/guardians had submitted signed consent forms. The researchers administered the instruments in classrooms and verified the inclusion criteria for the study: (a) understanding of the Italian language; (b) absence of serious or severe intellectual pathologies; (c) absence of auditory and visual deficits. Study procedures were implemented during class time and anonymity of the participants was ensured. The study involved an assessment of suggestibility and autobiographical narrative skills.

### 2.2 Procedure

The instruments were administered following the same procedure with all the participants: the Gudjonsson Suggestibility Scale 2 (GSS2) was administered first, while the autobiographical memory instrument was administered during the latency phase between the memory task and the suggestive interview of the GSS2.

Immediate recall of the GSS2 and autobiographical memory were collected in written form by the participants themselves; while the suggestive interview was administered orally and individually as required by the standard test procedure (Gudjonsson, 1997).

The study was conducted following and respecting the ethical principles in accordance with ethical research involving children. The study conformed to all ethical guidelines for research with human participants and followed the Declaration of Helsinki. The informed consent was signed before the inclusion of the children in the study, and it contained information on the objective of the study, methods of conduct, anonymity, and information on the conservation of sensitive data. The study was approved by the institutional ethics committee.

### 2.3 Instruments

#### 2.3.1 Gudjonsson Suggestibility Scales

The Gudjonsson Suggestibility Scale 2 (GSS2; Gudjonsson, 1997) is a validated instrument for measuring suggestibility levels in children aged 7 to 16 (Gudjonsson et al., 2016). The Italian GSS2 has good reliability and internal consistency (Cronbach’s alpha coefficient: Yield 1, α = 0.81; Yield 2, α = 0.83; Shift, α = 0.71; and Total Suggestibility, α = 0.77; Gudjonsson et al., 2016). The Italian version has already been used in several studies (Vagni et al., 2015; Gudjonsson et al., 2020, 2021, 2022).

The administration of the scales involves the reading of a short story asking participants for the Immediate Recall (IR) whose maximum score is 40 items. After 50 min of latency, the participants experience the interview which includes 15 leading questions (for example: “Did the boy’s bicycle get damaged when it fell to the ground?” and “Did John grab the boy’s arm or shoulder?”) and five neutral questions (“Were the couple called Anna and John?”). After the interview a negative feedback is given (“You have made some mistakes. Now I repeat again the questions. Please try to be more accurate”) and the same interview are administrated again. The sums of the acceptance responses to the leading questions at the first and second interviews are Yield1 and Yield 2, respectively. The number of changed answers after the negative feedback is Shift score. The Total Suggestibility score is given by the sum of Yield1 and Shift.

#### 2.3.2 Children Recalling Autobiographical Memory

The Children Recalling Autobiographical Memory (CRAM) is a tool that includes two session, tapping into either retrospective or prospective autobiographical memory skills, as they both refer to similar cognitive processes and functions. In each session, 10 cue-words are presented in relation to which the children are asked to recall a personal event with the following instruction: “*Below you will find a series of words, for each one try to remember (or imagine in the prospective memory section) an event in your life that has happened, specifying as far as possible where, what, how, when and with whom it happened*.” Similar instructions were used in the semi-structured autobiographical questionnaire by Piolino et al. (2002) albeit without the cueing technique.

The participants received the CRAM test in paper form through a small booklet where the delivery and sequence of the cue words were reported, interspersed with a large space to allow the autobiographical memories to be reported in written form. The last section was related to questions that probe semantic autobiographical memory.

The instrument’s stimuli can be organized into the following categories: General Time; Specific Events; Places; Common Objects; and Emotions.

In general, imageable, concrete, and meaningful words cued autobiographical memories more often and faster and cued also older autobiographical memories (Rubin and Schulkind, 1997).

The analysis of previous studies on autobiographical memory has suggested the selection of some cue words, such as those relating to basic positive and negative emotions (such as: Joy, Fear and Sadness), already used with children (Uzer et al., 2012; Nieto et al., 2017; Ros et al., 2018). We included also the stimuli used in the validation of an instrument on the autobiographical memory of elderly people (Pierucci et al., 2023). For the General Time category, days of the week and seasons were used as in the study by Bauer et al. (2007). This study also used names of concrete objects to recall autobiographical events included in our Objects category. For the category of Specific Events, cue words already used in other studies with children were used, such as: birthday, New Year’s, party and wedding (Hudson and Fivush, 1991; Piolino et al., 2002).

A previous pilot study with child participants was conducted to test the cue words used (Vagni et al., 2023), and a good reliability index had been obtained (Cronbach’s *alpha* of 0.82). The lack of or poor response to some items suggested the replacement of some terms such as “Train” was changed to “Airplane.”

According to several studies, the main narrative skills refer to the ability to report space-time information (Nelson and Fivush, 2004), relating to the description of what happened, the people involved and how a certain episode occurred (Rubin et al., 2003; Wood and Conway, 2006). The test, therefore, detects 5 main narrative skills: Where, What, When, Who, and How; for each of them, 1 point is assigned in the scoring when it is present in the recall. It follows that for each cue-word it is possible to obtain a score from 0 to 5. In identifying a quantification method that is as objective as possible, the criteria of Levine et al. (2002) were followed who assign a score of 1 to each of the categories covered. For example, a child responded to the cue word joy: “I went to Rome with my parents and took some photos in front of the Colosseum.” The answer satisfied the category “where,” “what” and “who,” but not “when” and “how.” The final score awarded was 3.

To the cue word “school” a 13-year-old boy replied: “Two weeks ago I was in the school corridor talking to a friend. Marco arrived and stood in front of me, he quickly raised his hand and slapped me on the cheek and telling me I should not laugh at him in class anymore.” The answer satisfies all the categories of “when,” “where,” “what,” “who” and “how” obtaining a score of 5.

The tables in the appendices show the cue words used and the scoring sheet.

The scoring of the memories collected by the children was done by two independent evaluators. Inter-rater reliability was performed using Cohen’s K (K = 0.766; *p* < 0.001).

## 3 Data analysis

To validate the CRAM questionnaire, we conducted an exploratory factor analysis (EFA) in order to assess its factor structure in the tested sample. Before conducting this analysis, we checked its assumptions by performing the Kaiser–Meyer–Olkin test (KMO) and the Bartlett’s test of sphericity. In order to individuate the best number of factors to be extracted with EFA, we inspected the scree plot and analyze the eigenvalues of a solutions including 1 to 20 factors, following the Kaiser–Guttman’s criterion and then extracting factors with eigenvalue greater than 1.0. The EFA was conducted with maximum likelihood extraction method and with no rotation, as we have no expectation about numbers and correlations among factors. Then, the factors individuated in the EFA were inspected at item level and their internal validity assessed by means of Cronbach’s alpha, McDonald’s omega, and item-score correlation. The selected model was than evaluated using four fit indices: relative chi-square (χ^2^/df), comparative fit index (CFI), the Tucker–Lewis index (TLI), and the root mean square error of approximation (RMSEA). We assumed as a good fit relative χ^2^ ≤ 3.00, CFI ≥ 0.90, TLI ≥ 0.90, and RMSEA ≤ 0.05 (Hu and Bentler, 1999).

After this first analysis, we inspected the relationship between the demographic variables on CRAM and GSS2 score, and the relationship of the CRAM score and the suggestibility as measured by the GSS2 scores. We conducted this analysis by means of Pearson’s bivariate correlations, for age, CRAM score, and GSS2 scores, and by means of Student’s t-tests for the effect of gender.

Finally, we investigate the relationship of autobiographical skills with suggestibility while controlling for demographic variables. To do so, we conducted a multiple linear regression model in which the antecedent variable was the CRAM score, the covariates age and gender, and the dependent variables the GSS2 score. As we obtained a significant relationship for both age and CRAM score with GSS2 scores, we finally tested if the interactions of those two variables significantly modify the relationship between them and suggestibility, by adding a further level of computation to the regression model.

### 3.1 Results

**Figure 1:**
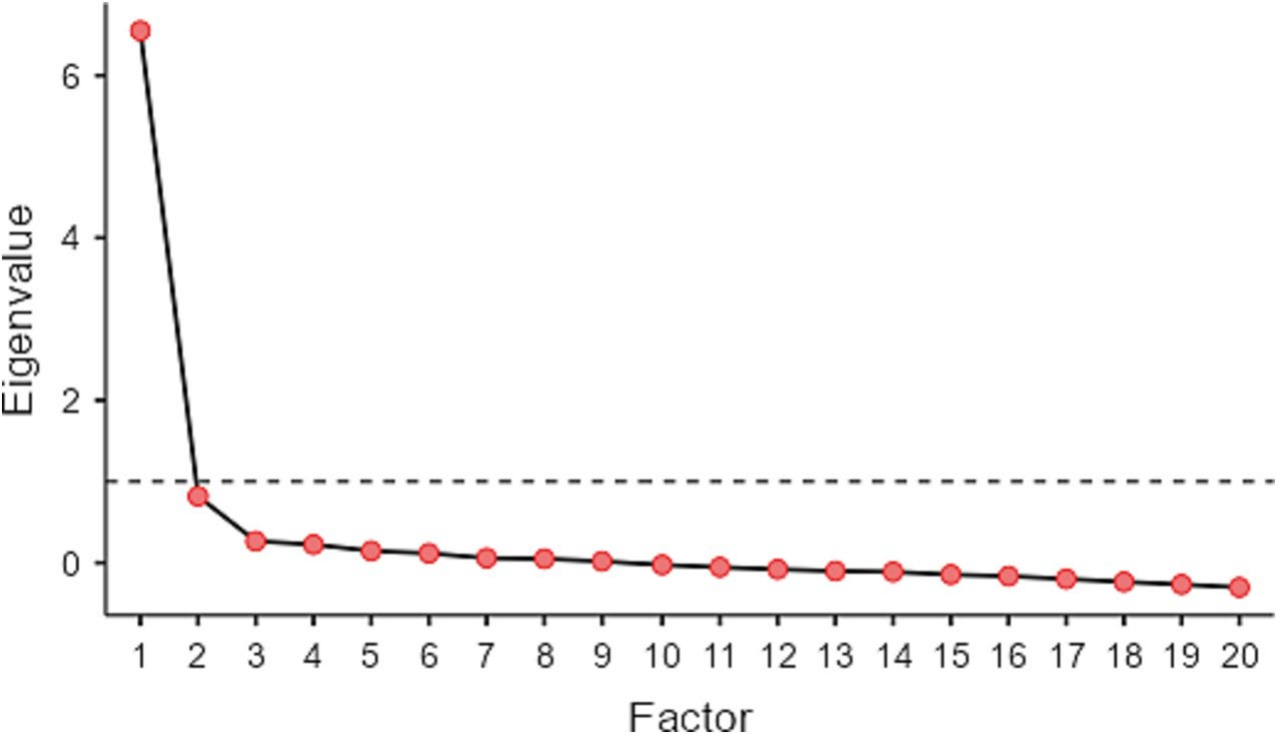
Scree plot of the eigenvalues for the factor 1 to 20. Dotted line represents the eigenvalue = 1. As reported, only the first factor reveals an eigenvalue higher than 1.

The CRAM items were checked for EFA assumptions. The KMO test revealed an overall value of 0.94, and the Bartlett’s test of sphericity was significant, *χ^2^*(190) = 2,071, *p* < 0.001, indicating that assumptions for the EFA analysis were met. The analysis of the eigenvalues and of the scree plot (see Figure 1) support a single-factor solution. In particular, following the Kaiser–Guttman’s criterion, only the first factor had an eigenvalue higher than 1.0 (6.55), whereas the other had lower eigenvalues (<0.82). Therefore, we further conducted the EFA with a single factor.

The result of the EFA analysis was reported in Table 1, along with items’ scores (mean and SD) and factor loadings. The one-factor solution adopted explained about 33% of the total variance and all items showed loadings >0.429. The one-factor model revealed also good fit indexes, with relative *χ^2^* = 1.93, *CFI* = 0.92, *TLI* = 0.91, and *RMSEA* = 0.05 [0.04, 0.06]. Based on this analysis, we computed an overall score for autobiographical memory to be used in the subsequent analyses.

**Table 1:** Factor loadings of EFA and descriptive statistics for the CRAM items.

Item	Original item	Factor loading	Mean	SD
Bike	BICI	0.65	2.45	1.12
Birthday	COMPLEANNO	0.67	2.61	1.12
Ball	PALLA	0.64	2.11	1.22
Mountain	MONTAGNA	0.62	1.35	0.95
Night	NOTTE	0.62	1.57	0.96
New Year’s	CAPODANNO	0.61	2.43	1.13
Beach	SPIAGGIA	0.60	1.64	0.96
Summer	ESTATE	0.59	2.08	1.04
Joy	GIOIA	0.59	2.64	1.16
Party	FESTA	0.59	1.79	1.00
Airplane	AEREO	0.58	1.31	0.94
Home	CASA	0.58	2.14	1.17
Sadness	TRISTEZZA	0.57	2.22	1.19
Sunday	DOMENICA	0.54	2.40	1.16
Wedding	MATRIMONIO	0.54	1.10	0.98
Mobile phone	CELLULARE	0.53	1.47	0.86
School	SCUOLA	0.53	2.47	1.15
Fear	PAURA	0.48	1.45	0.90
Surprise	SORPRESA	0.44	1.46	0.92
Winter	INVERNO	0.43	3.16	1.12

First, we addressed the internal consistency of this score by conducting a reliability analysis. The CRAM score showed good internal reliability, with Cronbach’s *alpha* of 0.905 and McDonald’s *omega* was 0.906. Item-score correlations ranged from 0.412 and 0.622. Overall, this analysis supported a good level of reliability for the CRAM score.

We then move to the analysis of the relationship between the CRAM score, the GSS2 scores, and the demographic variables, i.e., age and gender. First, we tested the effect of gender on the psychological variables in a series of independent samples t-tests. As reported in Table 2, we obtained a significant effect for the effect of gender on CRAM score, with mean score for male = 35.30 (*SD* = 12.00) and mean score for females = 41.60 (*SD* = 14.50), whereas gender had no significant effect on any GSS2 scores, all *ps* > 0.219.

**Table 2:** Independent sample *t*-tests results with gender as independent variable (*N* = 321).

Variable	Males		Females				
	*M*	*SD*	*M*	*SD*	*Statistic*	*p level*	*Cohen’s d*
CRAM	35.50	12.00	41.60	14.50	−4.078	< 0.001	−0.456
TOT Sugg.	12.75	5.28	13.12	5.43	−0.612	0.541	−0.069
YIELD1	7.39	3.59	7.88	3.48	−1.233	0.219	−0.140
YIELD2	8.05	3.66	8.49	3.77	−1.054	0.293	−0.119
SHIFT	5.36	2.89	5.24	3.44	0.337	0.736	0.038

M, mean; SD, standard deviation; CRAM, Children Recalling Autobiographical Memory; TOT Sugg., Gudjonsson Suggestibility Scales total score. Cohen’s d indicates the effect size.

**Figure 2:**
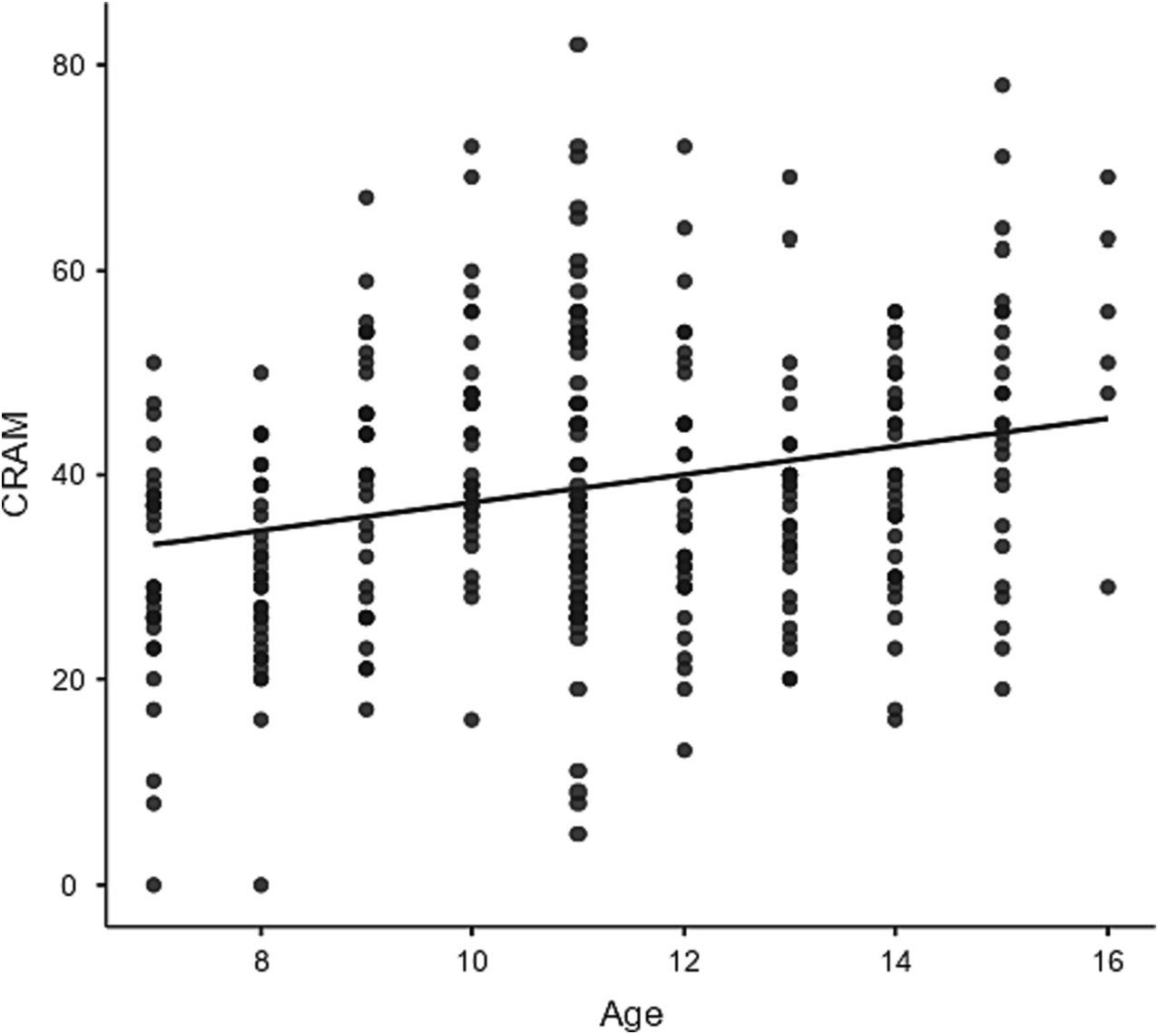
Scatterplot for the relationship between age and CRAM score. The continuous line represents the linear regression.

Afterward, we moved to test the correlations among age and the psychological variables, as reported in Table 3. Age was strongly and positively correlated with CRAM score (as depicted in Figure 2), and negatively correlated in a weaker manner with suggestibility scores, i.e., total GSS2 score, YELD1 and YELD2. In line with our hypothesis, CRAM score was negatively and mildly correlated with some suggestibility scores, such as YELD1 and total GSS2 score. Lastly, all the GSS2 scores were strongly and positively correlated with each other. Overall, this analysis indicated that the relationship between autobiographic skills and suggestibility was supported in our sample, and that age seemed related to both these variables. In a more refined analysis on the relationship between age and CRAM score, we decomposed the total CRAM score into the five aspects as related to What, Where, When, Who, and How, and correlated each of these sub-scores with the age. We found that age positively correlated with the scores When (*r* = 0.217, *p* < 0.001) and Who (*r* = 0.130, *p* < 0.05), and less strongly with Where (*r* = 0.108, *p* < 0.05) and How (*r* = 0.108, *p* < 0.05) scores, while it did not significantly correlate in our data with the What score (*r* = 0.071, *p* = 0.206).

**Table 3:** Descriptive statistics and correlation coefficients among age and psychological variables (*N* = 321).

Variable	Mean	SE	Age	CRAM	IR	YIELD1	YIELD2	SHIFT
Age	11.03	0.14	—					
CRAM	38.71	0.76	0.249^***^	—				
IR	13.48	0.33	0.133^*^	0.320^***^	—			
YIELD1	7.65	0.20	−0.197^***^	−0.166^**^	−0.306^***^	—		
YIELD2	8.28	0.21	−0.148^**^	−0.068	−0.153^**^	0.800^***^	—	
SHIFT	5.29	0.18	−0.019	−0.096	−0.208^***^	0.267^***^	0.400^***^	—
TOT Sugg.	12.95	0.3	−0.141^*^	−0.166^**^	−0.326^***^	0.819^***^	0.766^***^	0.771^***^

SE, standard error; CRAM, Children Recalling Autobiographical Memory; IR, Immediate Recall of the Gudjonsson Suggestibility Score; TOT Sugg., Gudjonsson Suggestibility Scales total score. Significance level is marked are follows: ^*^p < 0.05, ^**^p < 0.01, ^***^p < 0.001.

**Figure 3:**
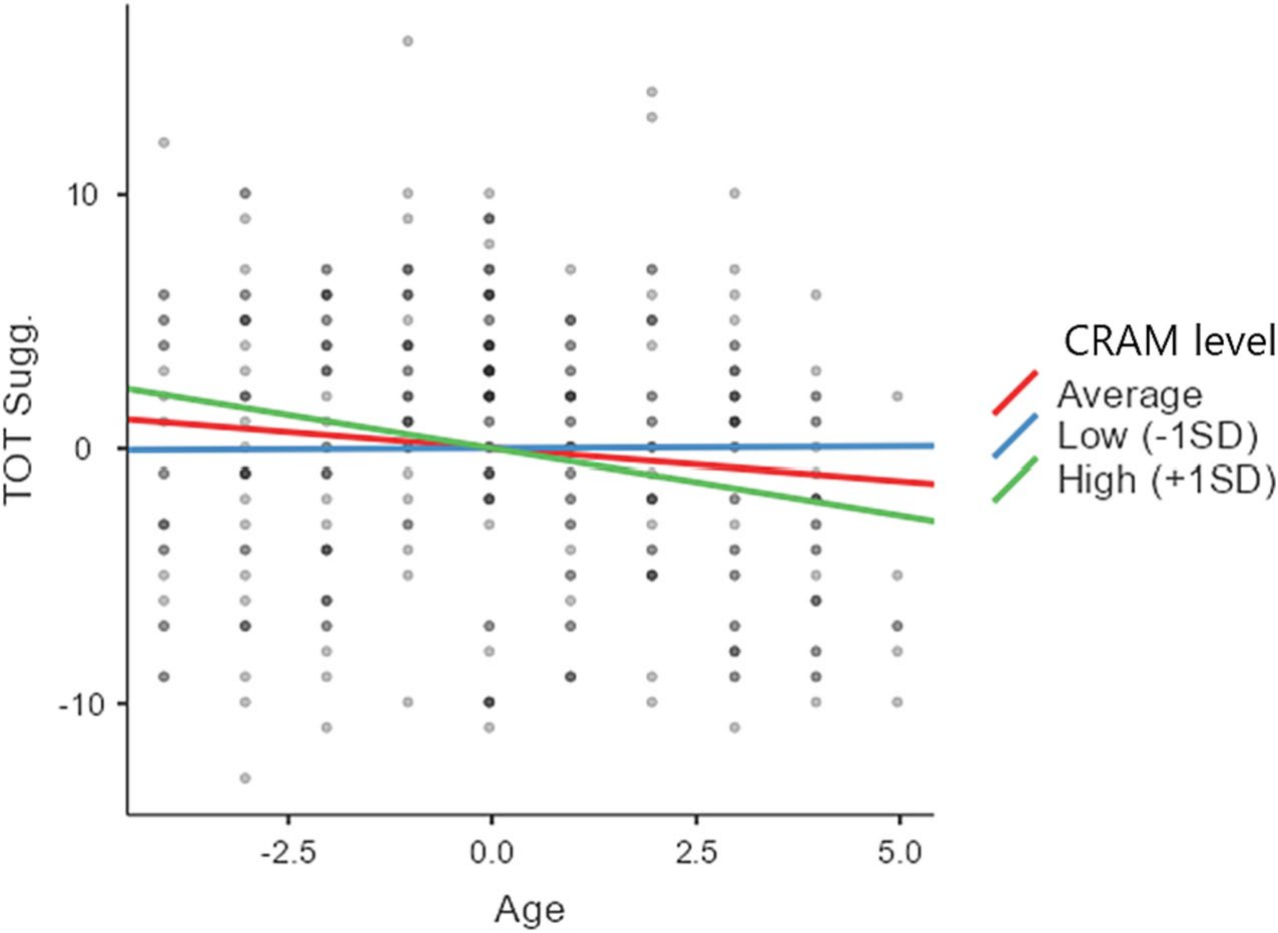
Simple slope analysis for the interaction between age and CRAM score on TOT suggestibility score of GSS2. Please notice that variables are mean centered.

Lastly, we investigate the hypothesis that the reduction of suggestibility observed with age is mainly due to an increase in autobiographical skills. Based on the previously reported results, we than conducted a hierarchical linear regression model, in which we included in the first step CRAM score and age as antecedent variables, gender as a covariate, and GSS2 total score as the dependent variable, while in the second step we added the interaction term CRAM score * age. On note, removing the covariate gender did not change the pattern of results obtained for the other variables. The first step model was significant, *F*(3, 309) = 4.92, *R* = 0.214, *R^2^* = 0.046*, p* < 0.01. Model estimates and statics are reported in Table 4, including both regression coefficients (as *b*) and standardized coefficients (as *β*). As reported, both age and CRAM score had a significant negative relationship with suggestibility, while age had no relationship with the dependent variable. The second step model also included the interaction term CRAM * age. Adding this term significantly increased model fit, *ΔR^2^* = 0.016, *p* < 0.022. In fact, as reported in Table 4, the interaction term was significant. To further probe this interaction, we ran a simple slope analysis on the effect of age on suggestibility at three levels of autobiographical skills, i.e., low (−1 *SD* from the mean), medium (mean value), or high (+1 *SD* from the mean). We obtained a non-significant relationship of age and suggestibility when CRAM score was low, *b* = 0.016, *SE* = 0.168, *p* = 0.925, whereas such a relationship was significant for average, *b* = −0.253, *SE* = 0.121, *p* = 0.037, or high, *b* = −0.522, *SE* = 0.174, *p* = 0.003, level of CRAM score. The slopes are also reported in Figure 3. Overall, this analysis indicates that autobiographical skills are related to suggestibility irrespective of age and that they could also moderate the effect of age on suggestibility, meaning that only children with medium or high levels of those skills are less likely to be susceptible to external influences.

**Table 4:** Hierarchical regression results.

			95% confidence interval				
Predictor	b	SE	Lower	Upper	t	*P*	β
**STEP 1**							
Intercept	16.811	1.631	13.601	20.022	10.30	<0.001	
Age	−0.252	0.125	−0.499	−0.006	−2.02	0.045	−0.115
Gender	0.898	0.612	−0.307	2.102	1.47	0.144	0.084
CRAM	−0.062	0.022	−0.107	−0.018	−2.74	0.006	−0.160
**STEP 2**							
CRAM * age	−0.021	0.008	−0.038	−0.003	−2.31	0.022	−0.127

b, regression coefficients; SE, standard error; β, standardized coefficients; CRAM, Children Recalling Autobiographical Memory.

## 4 Discussion

The main objective of the study was to validate the CRAM tool. The EFA analysis conducted on the CRAM items indicated good fit indices supporting a single-factor solution. The one-factor solution adopted explained about 33% of the total variance. The internal consistency was assessed by conducting a reliability analysis. Overall, this analysis supported a good level of reliability for the CRAM score (see Table 1). These results confirm the application of the theoretical model of Conway and Pleydell-Pearce (2000) and Conway et al. (2004) according to which retrospective and prospective memory are similar and constitute autobiographical memory. The one-factor solution is in line with other tools that measure autobiographical memory (Griffith et al., 2012; Nieto et al., 2017).

The validation of a metric instrument for assessing autobiographical narration skills in children of different age groups represents a critical step in achieving an objective and comparable measure of autobiographical memory, particularly in the context of forensic applications.

Our results revealed a significant correlation between age and the total CRAM score, consistent with the existing literature’s understanding that autobiographical narration skills tend to improve as children grow older. It is well-established in the literature that children typically begin to develop a more precise temporal understanding of events after reaching the age of 10. While the ability to describe “What” appears to be present even in preschool-aged children, the capacity to describe “How” seems to require more advanced cognitive abilities. We also found difference in autobiographical skills based on gender. In fact, an independent sample t-test showed higher scores in females in the ability to recall autobiographical events. This result is in line with other studies that have found that females tend to present both specific and general autobiographical memories that are richer in detail (Rubin, 2000; Wang et al., 2011). However, no differences related to gender were obtained with respect to suggestibility scores (see Table 2). According with Gudjonsson (2003), suggestive vulnerability does not seem to be linked to gender but to the age factor. In our sample, age presented significant positive correlations with the Immediate Recall of the Gudjonsson Suggestibility Scale and the CRAM total score, and negative correlations with the GSS2 scores. As children grow up, they tend to develop greater semantic and autobiographical narration skills, and resistance to suggestibility factors, especially those of a cognitive nature linked to Yield 1 and 2 (Gudjonsson, 2003; Vagni et al., 2023). The vulnerability to criticism and to the socio-emotional pressures involved in the suggestive interview would tend to remain constant and would seem to be linked to factors independent of mnemonic and source monitoring abilities (Gudjonsson, 1987, 2003; Gudjonsson et al., 2016; Vagni et al., 2023).

The hierarchical linear regression model provides strong support for the idea that the decrease in suggestibility observed with age may be primarily attributed to an increase in autobiographical skills (as depicted in Table 4). The results underscore how both the overall autobiographical memory score and age exhibit a negative predictive influence on total suggestibility. Importantly, the mediating interaction between these two variables amplifies the reduction effect on total suggestibility scores (as observed in Step 2 of Table 4). Further simple slope analyses were conducted by categorizing the total autobiographical skills score into low, medium, and high categories. These analyses revealed that the interaction effect with age was irrelevant when the CRAM score was low. Conversely, the relationship between age and suggestibility is strong and negative in children with average or high levels of autobiographical skills (as illustrated in Figure 1). This result of our study seems consistent with that reported by Kulkofsky and Klemfuss (2008), according to which having high autobiographical narrative skills allows children to reject leading questions. In fact, children with greater narrative skills may have more confidence in themselves and their memory abilities associated with greater source monitoring which tends to increase with age (Gudjonsson et al., 2021, 2022).

High autobiographical narrative skills appear to reduce children’s vulnerability to the cognitive factors involved in suggestive interaction or interview. The ability to resist leading questions seems to be linked to greater source monitoring skills, intellectual and expressive abilities (Gudjonsson, 2003). In the study no significant association was found with the effects of negative feedback which leads children to change their initial responses. The shift in the theoretical construct of interrogative suggestibility is more associated with social and emotional pressures. Therefore, even children with high autobiographical abilities may be vulnerable to emotional and social pressures. This suggests that in forensic practice the joint objective measurement of both autobiographical memory and interrogative suggestibility might be appropriate.

The study is not without its limitations. Firstly, it is essential to acknowledge that the study’s concurrent validity is incomplete. In fact, we only conducted an EFA, while a confirmatory factor analysis (CFA) is missing. The inclusion of a CFA could provide additional confirmation regarding whether the one-factor solution we identified is the most suitable model for fitting this dataset. We recognize the potential significance of such analysis. Addressing this limitation, future studies employing diverse samples could shed further light on this matter. On another side, while the CRAM exhibits a positive correlation with the immediate recall of the GSS2, which involves the recall of a semantically learned task, no such correlation was explored with another autobiographical memory task or instrument, which limits our understanding of the CRAM’s validity in a broader context. Furthermore, the number of participants, although substantial, could be increased across different age ranges to enhance the study’s statistical power and the generalizability of the results in relation to their effects on suggestibility levels. Additionally, the study primarily focused on assessing the relationship between autobiographical memory and suggestibility without delving into the potential moderating or mediating variables that may influence this relationship. Future investigations could benefit from exploring such factors to provide a more comprehensive understanding of the mechanisms at play. The generalization of the results of this study to the forensic context may be limited as the stress factors that are normally activated in the forensic context and also the traumatic feelings and experiences that child victims and witnesses may experience could affect autobiographical abilities. This suggests that the association between CRAM and GSS2 should also be tested with child witnesses. Lastly, while the CRAM tool is a promising addition to the field, it still requires further validation and refinement to ensure its reliability in assessing autobiographical skills. In particular, further investigations appear necessary to understand the association between autobiographical memory and suggestive socio-emotional pressures during the recall phases.

## 5 Conclusion

Children’s testimony requires give close attention to their ability to recall autobiographical events and to refusal the suggestibility factors involved in a possible suggestive interview. Knowing how to tell an autobiographical event requires the child to be able to provide the main information on where, when, who, what and how. This information corresponds to the main autobiographical narrative skills that tend to increase with age. In this study, both autobiographical memory and immediate suggestibility were quantitatively assessed, offering valuable insights. Our analyses have demonstrated the validity of the Children Recalling Autobiographical Memory (CRAM) tool for measuring autobiographical skills in children aged 7 to 16. Furthermore, this study has highlighted that children with strong autobiographical skills exhibit greater resistance to immediate suggestibility factors, and this resistance appears to strengthen with age. These findings carry important implications, particularly in forensic practice. The CRAM test emerges as a valuable instrument for objectively measuring children’s autobiographical skills, with potential relevance in evaluating their vulnerability to suggestive influences in personal contexts. It is clear that enhancing our understanding of the relationship between autobiographical memory, suggestibility, and age contributes to more informed and responsible forensic practices when dealing with child testimonies.

## Data availability statement

The raw data supporting the conclusions of this article will be made available by the authors, without undue reservation.

## Ethics statement

The studies involving humans were approved by Comitato Etico per la Sperimentazione Umana—CESU of the University of Urbino. The studies were conducted in accordance with the local legislation and institutional requirements. Written informed consent for participation in this study was provided by the participants’ legal guardians/next of kin.

## Author contributions

MV: Conceptualization, Data curation, Formal analysis, Investigation, Methodology, Validation, Writing – original draft, Writing – review & editing. VG: Conceptualization, Data curation, Investigation, Methodology, Writing – original draft, Writing – review & editing. LS: Conceptualization, Data curation, Formal analysis, Investigation, Methodology, Validation, Visualization, Writing – original draft, Writing – review & editing.

## Appendix

**TABLE A1:** Cue-words of the CRAM test.

Category	Retrospective Memory	Prospective Memory
General Time	Winter/Sunday	Summer/Night
Specific Event	Birthday/New Year’s	Party/Wedding
Places	School/Home	Beach/Mountain
Objects	Bike/Ball	Mobile phone/Airplane
Emotions	Joy/Sadness	Surprise/Fear

**TABLE A2:** Scoring sheet of the CRAM test.

Cue-word	Recall	Where	What	When	Who	How	Tot.
Winter							
Joy							
School							
